# A Two-Step Feature Selection Method to Predict Cancerlectins by Multiview Features and Synthetic Minority Oversampling Technique

**DOI:** 10.1155/2018/9364182

**Published:** 2018-02-07

**Authors:** Runtao Yang, Chengjin Zhang, Lina Zhang, Rui Gao

**Affiliations:** ^1^School of Mechanical, Electrical and Information Engineering, Shandong University at Weihai, Weihai 264209, China; ^2^School of Control Science and Engineering, Shandong University, Jinan 250061, China

## Abstract

Cancerlectins have an inhibitory effect on the growth of cancer cells and are currently being employed as therapeutic agents. The accurate identification of the cancerlectins should provide insight into the molecular mechanisms of cancers. In this study, a new computational method based on the RF (Random Forest) algorithm is proposed for further improving the performance of identifying cancerlectins. Hybrid feature space before feature selection is developed by combining different individual feature spaces, CTD (Composition, Transition, and Distribution), PseAAC (Pseudo Amino Acid Composition), PSSM (Position-Specific Scoring Matrix), and disorder. The SMOTE (Synthetic Minority Oversampling Technique) is applied to solve the imbalanced data problem. To reduce feature redundancy and computation complexity, we propose a two-step feature selection process to select informative features. A 5-fold cross-validation technique is used for the evaluation of various prediction strategies. The proposed method achieves a sensitivity of 0.779, a specificity of 0.717, an accuracy of 0.748, and an MCC (Matthew's Correlation Coefficient) of 0.497. The prediction results are also compared with other existing methods on the same dataset using 5-fold cross-validation. The comparison results demonstrate the high effectiveness of our method for predicting cancerlectins.

## 1. Introduction

Lectins are a diverse group of proteins that exhibit relatively high affinity and specificity toward carbohydrate residues of glycoproteins and glycolipids [1]. They are ubiquitously present in living organisms, including viruses, bacteria, fungi, Protista, plants, and animals [2–4]. These sugar-binding proteins are generally classified in accordance with their carbohydrate specificities: mannose, galactose/N-acetylgalactosamine, N-acetylglucosamine, fucose, and sialic acid [5]. Due to their ability to recognize cell-surface carbohydrates with high specificity, lectins have been implicated in various essential biological processes, including cell-cell communication, cell proliferation, cell arrest, apoptosis, host-pathogen interactions, tissue development, and tumor cell metastasis [6]. Owing to the sugar-binding ability of lectins, they are basic tools in glycomic studies [7]. Several glycan structures that have been reported to change glycoproteins in different cancers can be targeted by certain plant lectins [8].

Cancer is a leading cause of death characterized by an abnormal and unregulated growth of cells. Although survival rates are improving for many types of cancer, new cancer drugs are still in high demand [9]. Cancerlectins are those lectins related to cancers. Cancerlectins have a protective effect against the growth of cancer cells [10]. They have the least side effects, which suggests the importance of developing antitumor drugs based on lectins [9]. Growing evidence has shown that they are currently being employed as therapeutic agents, resulting in cancer cell agglutination and apoptosis, thus impeding tumor progression [9, 10]. For instance, nagaimo lectin is worth exploring for the treatment of breast cancer [11]. Lectin from banana has been shown to inhibit HIV replication and thus is investigated as a treatment for AIDS [12]. Recurrent skin infections and certain forms of inflammatory skin disease may be caused by mannose-binding lectin deficiency [13]. Through triggering receptor-mediated signaling pathways, the legume lectins could induce cancer cell death [14]. Mistletoe lectin can inhibit cell growth and induct cancer cell apoptotic through triggering molecular changes [15]. Galectins have great potential in the treatment, prevention, and diagnosis of specific cancers by contributing to tumorigenesis, proliferation, angiogenesis, and metastasis [16–18].

Although most lectins are shown to possess antitumor properties, gaps between our knowledge about lectin biology and their interacting proteins still exist. It is beneficial for developing lectin-based drugs to clarify the molecular mechanisms underlying the biological effects of lectins [9]. Furthermore, the limited natural cancerlectins are difficult to fulfill the current requirements [7]. Therefore, the accurate identification of the cancerlectins should provide insight into the molecular mechanisms of cancers. The knowledge gained may provide a basis for improved diagnosis and treatment of many diseases. As the available cancerlectins are limited, the newly identified cancerlectins are of high value for advanced research in pursuing several applications in biotechnology, immunology, and clinical practice.

Experimentally identifying cancerlectins are time-consuming, tedious, and costly, especially for the rapid accumulation of protein sequences. In view of this, it is highly desired to develop automated high throughput computational methods for predicting cancerlectins. Traditional computational approaches for protein function prediction have explored homology relationships using the Basic Local Alignment Search Tool (BLAST) [19] program to associate the known function of its homologous with the query protein. As given in [20], BLAST achieves a poor prediction performance in distinguishing between cancerlectins and noncancerlectins. This may be due to the fact that lectins from tumor cells share marked sequence homology with lectins from normal tissues [21].

In the last few years, machine learning methods have attained the promising results for identifying cancerlectins. Kumar et al. [20] proposed the first computational program based on machine learning methods for the prediction of cancerlectins. They developed a Support Vector Machine (SVM) model incorporating the PROSITE domain information and Position-Specific Scoring Matrix (PSSM). Lin et al. [22] developed a sequence-based method to discriminate between cancerlectins and noncancerlectins. The *g*-gap dipeptide composition was employed to encode protein sequences. The proposed method achieved an accuracy of 0.752, which is superior to the method given in [20]. However, due to the imbalanced dataset, there is a great divergence between sensitivity (0.691) and specificity (0.801). In addition, Lin et al. [22] extracted features from protein sequences based on a single technique, which may limit the prediction performance. Generally, prediction performance can be enhanced through effectively combining feature extraction methods from different sources [23].

The aim of this work is to propose a new predictor for further improving the prediction performance of identifying cancerlectins. To fully extract information from the original sequence, four methods for feature extraction—namely, CTD (Composition, Transition, and Distribution), PseAAC (Pseudo Amino Acid Composition), PSSM (Position-Specific Scoring Matrix), and disorder—are employed to effectively transform the protein sequences into feature vectors. As the present dataset is imbalanced, we use SMOTE (Synthetic Minority Oversampling Technique) to balance the dataset. In order to reduce computation complexity and feature redundancy, a two-step optimal feature selection process is proposed to find the optimal feature subset. Based on the optimal feature subset, the prediction is carried out by the Random Forest (RF) classifier. Compared to previous studies [20, 22], our method achieves both a high sensitivity (0.779) and a high MCC (0.531) in 5-fold cross-validation. The results show that the proposed method is an improved and alternative method for identifying cancerlectins.

## 2. Materials and Methods

### 2.1. Datasets

To evaluate the performance of the proposed method and compare it with existing methods, a publicly available dataset [20, 22] is employed. After removing the proteins having 100% sequence similarity using CD-HIT [24], 385 cancerlectins are obtained from CancerLectinDB [25]. By searching the UniProt Database [26] with the keyword “lectin” and then removing sequences tagged with “similar”, “fragment”, “putative”, and “probable”, a negative dataset including 820 proteins is built. 71 sequences that are found to be common to cancerlectins and lectins are then removed from lectins. To balance the datasets, a total of 385 sequences are randomly selected from the lectin sequences. To avoid an overestimation of the predictive performance, the sequences with more than 50% sequence similarity to any other one are removed using CD-HIT [24]. As a result, the final dataset consists of 178 cancerlectins and 226 noncancerlectins. The details of the protein sequences in the dataset are available in Supplementary File 1.

### 2.2. Feature Extraction

For developing a powerful predictor, constructing a comprehensive and proper feature vector of proteins is an important step. Generally, an individual feature extraction strategy does not preserve enough discriminative information to distinguish different protein classes [27]. To improve the prediction performance, a good combination of feature extraction methods is needed. In developing high throughput tools for predicting various important protein attributes, many different descriptors to represent sequence samples have been developed and widely used. In this study, hybrid features extracted from CTD, PseAAC, PSSM, and disorder are utilized to transform the protein sequences into feature vectors.

#### 2.2.1. Composition, Transition, and Distribution

A global feature extraction strategy called Composition, Transition, and Distribution (CTD), introduced by Dubchak et al. [28], can effectively extract global information of protein sequences.

The 20 natural amino acids are divided into three groups, polar, neutral, and hydrophobicity groups, according to the seven physicochemical properties, hydrophobicity, normal Vander Waals volume, polarity, polarizability, charge, secondary structure, and solvent accessibility. Details about the division of the amino acids are given in Table 1.

For a given physicochemical property in Table 1, composition (*C*) describes the global percent composition of each of the three subgroups, which is defined as

(1) $$\begin{array}{c} \left(\;\frac{N_{1}}{L},\frac{N_{2}}{L},\frac{N_{3}}{L}\;\right), \end{array}$$

where *N*_*i*_, *i* ∈ {1,2, 3}, denotes the number of amino acids that belong to group *i* and *L* is the length of the given protein sequence.

Transition (*T*) characterizes the percent frequency with amino acids from one subgroup followed by amino acids from a different subgroup, which can be calculated by

(2) $$\begin{array}{c} \left(\;\frac{N_{\alpha_{1}\alpha_{2}}+N_{\alpha_{2}\alpha_{1}}}{L},\frac{N_{\alpha_{1}\alpha_{3}}+N_{\alpha_{3}\alpha_{1}}}{L},\frac{N_{\alpha_{2}\alpha_{3}}+N_{\alpha_{3}\alpha_{2}}}{L}\;\right), \end{array}$$

where *α*_*i*_, *i* ∈ {1,2, 3}, represents one of the amino acid groups. *N*_*α*_*i*_*α*_*j*__ is the number of the dipeptides encoded as “*α*_*i*_*α*_*j*_.”

Distribution (*D*) measures the respective locations of the first, 25%, 50%, 75%, and 100% of the amino acids within each subgroup, which is defined as

(3) N11L,N12L,…,N15L,N21L,N22L,…,N25L,N31L,N32L,…,N35L,

where *N*_*i*1_ is the chain length within which the first of the amino acids of group *i* is located. *N*_*i*2_, *N*_*i*3_, *N*_*i*4_, and *N*_*i*5_ measure the chain lengths within which the 25%, 50%, 75%, and 100% of the amino acids of group *i* are located, respectively.

Based on the seven physicochemical properties listed in Table 1, a 147-dimension CTD feature vector is generated for a protein sequence.

#### 2.2.2. Pseudo Amino Acid Composition

The concept of Pseudo Amino Acid Composition (PseAAC) was originally introduced by Chou for predicting protein cellular attributes [29]. According to the concept of PseAAC, a protein sequence can be represented by a 20 + *λ* dimension vector. The first 20 numbers represent the occurrence frequencies of 20 amino acids in a protein, and additional factors incorporate some of the sequence order information via various modes. PseAAC has been proved to be an extremely effective feature in the field of protein attribute predictions, such as protein solubility prediction [30], protein subchloroplast localization prediction [31], and antimicrobial peptides prediction [32]. The concept of PseAAC can be described as follows.

A given protein *P* with *L* amino acid residues is represented as

(4) $$\begin{array}{c} P=R_{1}R_{2}R_{3}\cdots R_{L-1}R_{L}, \end{array}$$

where *R*_1_ represents the first residue of the protein *P*, *R*_2_ represents the second residue,…, *R*_*L*_ represents the *L*th residue.

In the classical mode of PseAAC, a given protein *P* is formulated by a (20 + *λ*)-*D* vector as follows:

(5) $$\begin{array}{c} V={\left[\;v_{1},v_{2},\ldots ,v_{20},v_{20+1},\ldots ,v_{20+\lambda }\;\right]}^{T}, \end{array}$$

where

(6) $$\begin{array}{c} v_{u}=\left\{\begin{array}{cc} \frac{f_{u}}{\sum_{i=1}^{20}f_{i}+w\sum_{j=1}^{\lambda }\theta_{j}} & \left(1\leq u\leq 20\right)\\ \frac{w\sum_{j=1}^{\lambda }\theta_{u-20}}{\sum_{i=1}^{20}f_{i}+w\sum_{j=1}^{\lambda }\theta_{j}} & \left(20+1\leq u\leq 20+\lambda \right), \end{array}\right. \end{array}$$

and *f*_*u*_  (*u* = 1,2,…, 20) are the occurrence frequencies of the 20 native amino acids in the protein sequence *P*. The symbol *w* represents the weight factor for the sequence order effect, which ranges from 0.05 to 0.70. *θ*_*j*_ represents the *j*th tier sequence correlation factor calculated according to the following equation:

(7) θj=1L−j∑i=1L−j13H1Ri−H1Ri+j2+H2Ri−H2Ri+j2+MRi−MRi+j2,

where *H*^1^(*R*_*i*_), *H*^2^(*R*_*i*_), and *M*(*R*_*i*_) are standardized hydrophobicity, hydrophilicity, and side-chain mass of the *i*th amino acid of the protein sequence *P*.

Considering the fact that the lengths of the shortest protein sequence, *ω* and *λ*, are set to be 0.15 and 50, respectively, it is obvious that there are 70 features generated from PseAAC.

#### 2.2.3. Position-Specific Scoring Matrix

Evolutionary conservation, one of the most important aspects in biological sequence analysis, serves as an evidence for structural and functional conservation [33]. In the evolutionary process, functionally important region is always conservative [34]. Exploiting the detailed conservation pattern of residues is an effective way to facilitate the prediction of protein functions [35]. Evolutionary information in the form of PSSM [36] has been widely used to transform the variable lengths of protein sequences into fixed-length feature vectors.

For a protein sequence *P* with *L* residues, the PSSM profiles are generated by using the PSI-BLAST (Position-Specific Iterative Basic Local Alignment Search Tool) program [37]. Three iterative searches with a cutoff *E*-value of 0.001 are carried out against the UniProtKB/Swiss-Prot database.

The elements of PSSM are scaled to the range from 0 to 1 using the sigmoid function

(8) $$\begin{array}{c} f\left(\;x\;\right)=\frac{1}{1+e^{-x}}, \end{array}$$

where *x* denotes the original PSSM value.

PSSM-Amino Acid Composition (PSSM-AAC) aims to capture global discriminatory information related to the occurrence of each amino acid along a given protein sequence. PSSM-AAC is derived from the PSSM by summing the substitution score of each amino acid and divided by the total length of the protein, which is calculated as follows:

(9) $$\begin{array}{c} PSSM\text{-}AAC_{i}=\frac{1}{L}\overset{L}{\underset{n=1}{\sum }}E_{n\to i},\;\left(i=1,\ldots ,20\right), \end{array}$$

where *E*_*n*→*i*_ represents the score of the amino acid in the *n*th position of the query sequence being mutated to amino acid type *i* during the evolution process.

Pseudo PSSM, also called autocovariance (AC) method, is a powerful statistical tool developed by Wold et al. [38]. Pseudo PSSM gives the autocovariance of the substitution score of each amino acid along a protein sequence and is defined as follows:

(10) PsePSSMj,k=1L−j∑i=1L−jEi→k−Eave→kEi+j→k−Eave→k,k=1,2,…,20;  j=1,2,…,γ;  0<γ<L,

where *E*_ave→*k*_ is the average of substitution score of the amino acid *i* being mutated to amino acid type *k* along the whole sequence and *γ* is the autocorrelation coefficient. The value of *γ* is chosen as 5. Therefore, 20 × 5 = 100 features are calculated in this feature group.

The feature vector extracted from PSSM can be represented as

(11) $$\begin{array}{c} F_{\text{PSSM}}={\left[\;\begin{array}{cc} PSSM\text{-}AAC_{i} & PsePSSM_{j,k} \end{array}\;\right]}^{T}, \end{array}$$

where *T* denotes the transpose of the vector and *i* = 1,2,…, 20, *j* = 1,2,…, 5, and *k* = 1,2,…, 20.

#### 2.2.4. Disorder

A protein region is defined as “disorder” characterized by the lack of stable secondary or tertiary structure under physiological conditions or in the absence of a binding partner [39]. Since the disordered regions always contain sorting signals and allow for more modification sites, they carry out important roles in regulating protein functions, including enzyme catalysis, cell regulation, and ligand binding [40]. A number of studies have also reported that the incorporation of structural disorder improves the prediction performance [41, 42]. The disorder predictor “VSL2” [43] is employed to calculate the disorder score of each residue in a given protein.

The disorder score ranges from 0 to 1, where the higher the score is, the more likely the residue lacks a fixed structure. The following 28 features are designed to encode each protein sequence: (i) mean/standard deviation of all residues' disorder scores (2 features); (ii) number of disorder/nondisorder segments (2 features); (iii) minimum/maximum length of disorder/nondisorder segments (4 features); and (iv) the average disorder score of each native amino acid (20 features).

### 2.3. Synthetic Minority Oversampling Technique

The final dataset consists of 178 cancerlectins and 226 noncancerlectins, which leads to the imbalanced data classification problem, that is, high prediction accuracy for the majority class but poor prediction accuracy for the minority class. SMOTE (Synthetic Minority Oversampling Technique) is employed in this study to reduce the bias produced due to the unbalanced nature of data.

For oversampling the minority class, SMOTE selects a minority class sample and creates novel synthetic samples along the line segment joining some or all *k* nearest neighbors belonging to that class [44]. In this paper, to make the number of cancerlectins equal to the number of noncancerlectins, new cancerlectins in the feature spaces are generated via the SMOTE algorithm. Subsequently, this balanced dataset, having an equal number of cancerlectins and noncancerlectins, is used for training the predictor.

### 2.4. Two-Step Feature Selection

The original feature set generally contains redundant information or noise. Not all of the calculated features characterizing the protein sequence are relevant to the discrimination. Inclusion of redundant and noisy features would cause poor predictive performance and increased computation time [45]. In order to reduce feature redundant and computation complexity, we propose a two-step feature selection process to pick up informative features.

In the first step, we assess the feature vector elements using the Relief algorithm. Relief score is a good measure of the relevance of an attribute with respect to classes. For detailed description about the Relief score, please refer to [46]. According to this measure, the features then can be ranked by the Relief scores. Here, we select the top features with Relief score larger than 0.

In the second step, the wrapper-based method, SFS (Sequential Forward Selection), is employed to identify the optimal feature set from the top features ranked by Relief. More specifically, the procedure starts with an empty feature set and adds features one by one. A new feature set is constructed when another feature has been added. Each added feature is the one whose addition maximizes the prediction accuracy of the predictor. Repeat the process until all features have been added. The feature set that yields the highest cross-validation accuracy among all iterations is selected as the optimal feature set.

### 2.5. Random Forest

The Random Forest (RF) algorithm, developed by Breiman [47], has been successfully applied in the field of protein function predictions [48, 49]. RF is an ensemble classifier consisting of several decision trees. At each node, a subset of *m* out of the total *M* features is selected randomly and the most optimized split on these *m* features is employed to split the node. Combining multiple decision trees produced in randomly selected subspaces not only effectively reduces the correlation between trees but also prevents the overfitting problem. Each tree in the forest is grown to the largest extent possible without pruning. After constructing all trees, each tree yields a class label for a new object. The RF classifier will choose the class with the most votes over all trees.

The WEKA (Waikato Environment for Knowledge Analysis) [50] software package is used for the RF classifier, where default parameters are employed.

### 2.6. Performance Measures

In the statistical prediction, independent dataset test, subsampling (*K*-fold cross-validation) test, and jackknife cross-validation are often employed to examine the predictive capability of a predictor [51]. As elucidated by Eqs. 28–32 in [52] and demonstrated in a series of studies [53–56], among the 3 test methods, the jackknife cross-validation is deemed as the most objective one that can always yield a unique result and hence has been widely used to test the quality for various predictors. However, taking the size of the benchmark dataset into consideration, 5-fold cross-validation test is used in this study to reduce the computational time and compare with previous studies objectively. The whole dataset is randomly split into 5 nonoverlapping parts. Each part is used in turn as testing set with the remaining 4 parts as training set. This process is iterated 5 times to test each part, and measurements are calculated as the average values of the 5 testing subsets.

The performance of the prediction system is evaluated by the following measurements. These measurements are derived from four scalar quantities, TP, FP, TN, and FN, which represent true positive (correctly predicted cancerlectins), false positive (noncancerlectins incorrectly predicted as cancerlectins), true negative (correctly predicted noncancerlectins), and false negative (cancerlectins incorrectly predicted as noncancerlectins), respectively:

(12) Sn=TPTP+FN.Sp=TNTN+FP,Acc=TP+TNTP+FP+TN+FN,MCC=TP×TN−FP×FNTP+FNTP+FPTN+FPTN+FN.

Sensitivity (Sn) measures the proportion of the known cancerlectins that are correctly predicted as cancerlectins and specificity (Sp) measures the proportion of the known noncancerlectins that are correctly predicted as noncancerlectins. Accuracy (Acc) is the percentage of correct prediction for all samples. Matthew's Correlation Coefficient (MCC) is usually regarded as a balanced measure ranging from −1 to 1, with larger values standing for better prediction performance.

To further evaluate the performance of the proposed method, we also use the receiver-operating characteristic (ROC) curve. The ROC curve, one of the most reliable approaches in evaluating performance of classifiers [57], is obtained by plotting sensitivity on the *y*-axis against 1 − specificity on the *x*-axis. The ROC curve can be quantified by the area under the curve (AUC), which is a reliable measure for the performance measurement.

## 3. Results and Discussions

### 3.1. Comparison between Random Forest and Other Machine Learning Classifiers

To investigate the advantage of the RF method, the prediction performance of the RF method is compared with that of several state-of-the-art classifiers within the field of protein function predictions such as AdaBoost, DT (Decision Table), NNA (Nearest Neighbor Analysis), LR (Logistic Regression), NB (Naïve Bayes), and RBFNetwork.  Table 2 lists the prediction performance of these considered methods on the full features using 5-fold cross-validation. As shown in Table 2, the RF method yields the highest accuracy of 0.699 and the highest MCC of 0.398. The sorted order of classifiers according to the sensitivity is (i) NNA, (ii) RF, (iii) AdaBoost, (iv) DT, (v) RBFNetwork, (vi) LR, and (vii) NB. The specificity of the RF method (Sp = 0.695) is very close to that of NB (Sp = 0.699), but it is significantly better than that of AdaBoost (Sp = 0.540), DT (Sp = 0.540), NNA (Sp = 0.584), LR (Sp = 0.558), and RBFNetwork (Sp = 0.491), respectively. RF obtains a better trade-off between specificity (0.704) and sensitivity (0.695). These comparison results indicate that the RF method is superior to other machine learning methods in cancerlectin prediction.

**Figure 1:**
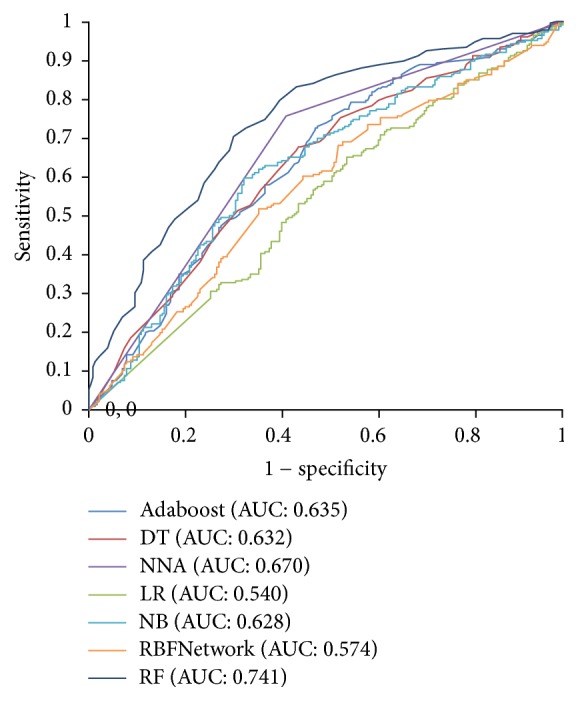
ROC curves of different machine learning classifiers. DT: Decision Table, NNA: Nearest Neighbor Analysis, LR: Logistic Regression, NB: Naïve Bayes, RF: Random Forest, and AUC: Area under the ROC curve.

Moreover, the ROC curves used for the assessment of the performance of these classifiers are plotted in Figure 1. We also calculate the AUC values for each classifier (Figure 1). The larger the value of AUC is, the better the performance of the model will be. Comparing with the other machine learning classifiers from Figure 1, the AUC (0.741) of the RF method covers the largest domains. Therefore, RF is an ideal choice among different machine learning methods to construct the optimal model for predicting cancerlectins.

### 3.2. Performance of the Current Method with or without SMOTE

In this section, the classification results of 5-fold cross-validation on the full features with SMOTE are compared with those without SMOTE. As shown in Table 3, without SMOTE, we achieve an Sp value as high as 0.717 but an Sn value as low as 0.461. The overall prediction results with SMOTE are significantly higher than those without SMOTE. The values of Sn, Acc, and MCC reach 0.704, 0.699, and 0.398, respectively, far better than the training results without SMOTE. These results provide strong evidence that SMOTE has an effective role in the performance of the proposed prediction system and it does solve the imbalanced data classification problem.

**Figure 2:**
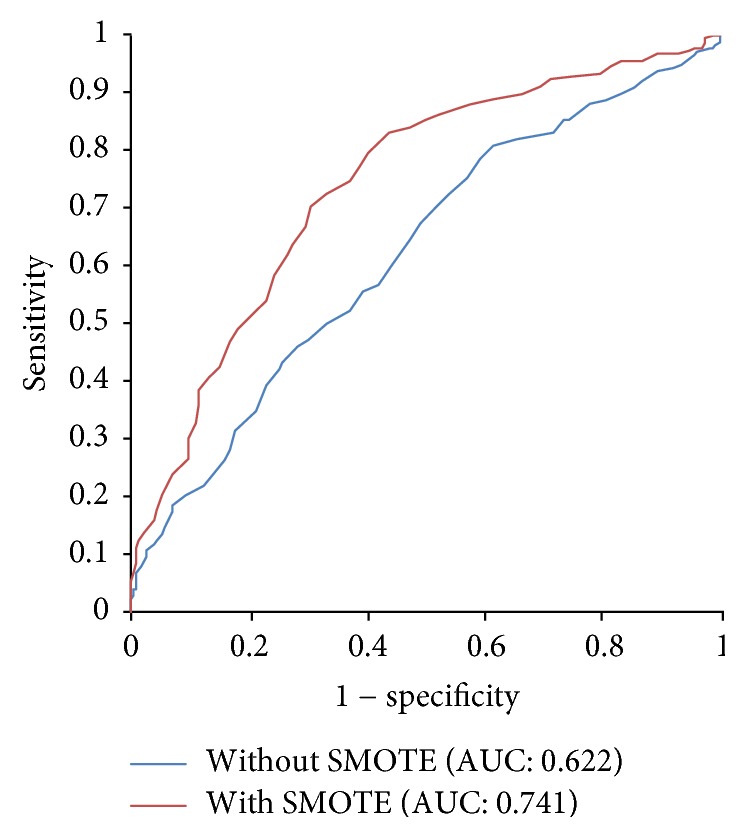
ROC curves with and without SMOTE on the full features using 5-fold cross-validation.

In addition, we perform ROC analysis to further compare the prediction performance with and without SMOTE. AUC is calculated with or without SMOTE as shown in Figure 2. The curve of the model with SMOTE is closer to the left side of the chart, primarily because it has high specificity values at all the thresholds compared to the model without SMOTE. The AUC of the model with SMOTE is about 0.741, which is significantly higher than the AUC (0.622) achieved by the model without SMOTE, indicating that SMOTE is truly very powerful.

### 3.3. Feature Selection Results

**Figure 3:**
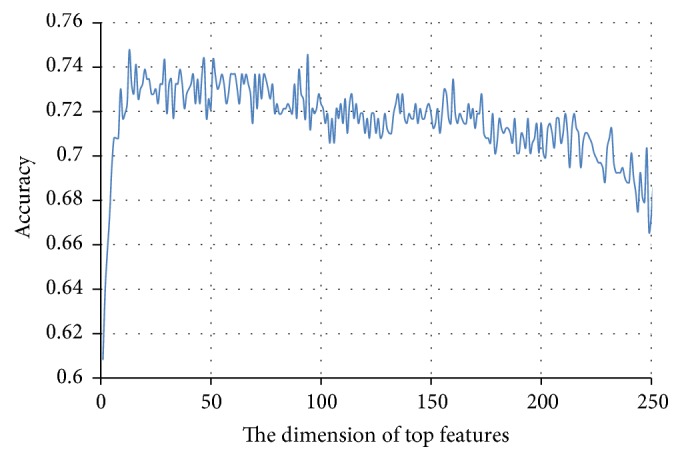
The prediction accuracy against the dimension of top features by performing the SFS (Sequential Forward Selection) scheme.

After running each feature extraction method, all primary protein sequences with different lengths are converted into 365 descriptors. Feature selection is then performed to pick out informative features from the 365 descriptors for the prediction of cancerlectins. Two stages are utilized in the feature selection process: (1) feature rank using Relief and (2) feature selection using the wrapper-based method. In the first stage, top 258 features with Relief score larger than 0 are selected. The score for each of the 365 descriptors evaluated by Relief is given in Supplementary File 2. In the second stage, feature selection is performed limited to this subset that is composed of 258 important features. The feature set that leads to the highest prediction accuracy is selected by performing the SFS scheme. The detailed prediction results against different numbers of features can be found in Supplementary File 3. With the number of features as *x*-axis and overall accuracy as the *y*-axis, the relation between the performance of the predictor and the feature subset is shown in Figure 3. The peak of the curve appears with the accuracy of 0.748 when the feature set is comprised of the first 13 features. The predictor thus trained by the 13 optimal features is used to identify cancerlectins. Of all selected features, 6 out of 13 features are extracted from PSSM, 5 out of 13 features are extracted from CTD, and 2 out of 13 features are extracted from PseAAC. We strengthen that the high quality of the proposed method is attributed to the combination of the selected features. In addition, the disorder based features are not selected in the final model. This is may be due to the fact that there are few features extracted from disorder.

### 3.4. Effectiveness Analysis of Feature Selection

**Figure 4:**
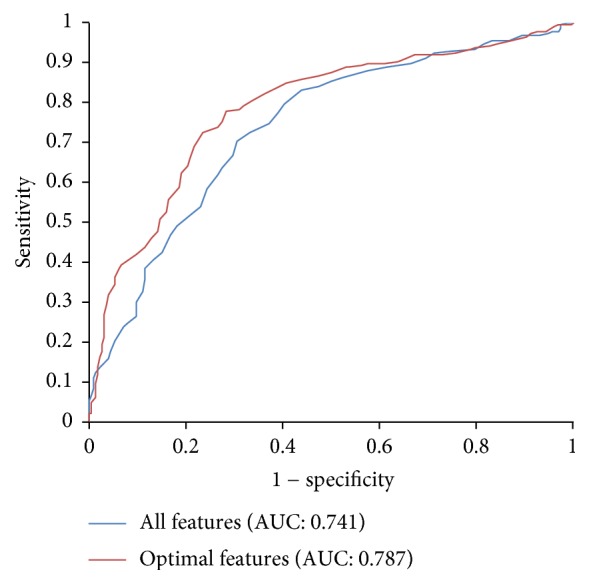
ROC curves for the classifiers using all the features and the 13 optimal features.

We investigate the effectiveness of the feature selection by plotting ROC curves for the classifiers using all the features and the 13 optimal features, respectively. From Figure 4 one can see that the AUC value with feature selection is significantly better than the AUC value without feature selection. The AUC for all features is 0.741 and for the top 13 features is 0.787, respectively. It appears that there is a substantial level of noise in the original feature set due to the existence of redundant or uninformative features. The two-step feature selection process employed in this study can significantly remove these redundant or uninformative features, thereby greatly improving the prediction performance of the model.

In order to further evaluate the effectiveness of the two-step feature selection method, randomly select 13 features from original features while keeping the class memberships unchanged. Then the prediction results are evaluated on the generated 13 features using the 5-fold cross-validation. This procedure is carried out 10 rounds. The averaged prediction performance is listed in Table 4 and compared with that obtained from optimal features. As can be seen from Table 4, the sensitivity, specificity, accuracy, MCC, and AUC of the optimal features are all superior to those of the randomly selected features. Therefore, it can be concluded that the two-step optimal feature selection method is effective and reliable.

### 3.5. Comparison with Other Methods

To further evaluate the prediction performance of the current method objectively, we make comparisons with some previously published methods on the same dataset using 5-fold cross-validation. The detailed results are illustrated in Table 5. As shown in Table 5, the proposed method has the highest sensitivity of 0.779 and the highest MCC of 0.497. The overall accuracy of the proposed method is only slightly lower than that of *g*-gap dipeptides [22] and exceeds other methods. The sensitivity of the current approach is 0.779, which is 0.088 higher than that of [22]. The high accuracy (0.752) and specificity (0.801) achieved by *g*-gap dipeptides [22] are notably accompanied with a low sensitivity (0.691). On the contrary, our method has a relatively balanced performance in terms of sensitivity (0.779) and specificity (0.717).

It is also important to highlight that the feature vector dimension of the proposed method is lower than those of the previous methods, which can reduce the computation complexity. These results demonstrate that the proposed method is superior to the previous studies and at the same time reduces the number of features used for this task significantly. As demonstrated in a series of recent publications [58, 59] in developing new prediction methods, user-friendly and publicly accessible web-servers will significantly enhance their impacts, and we shall make efforts in our future work to provide a web-server for the prediction method presented in this paper.

## 4. Conclusions

In this paper, we have developed a computational method to identify cancerlectins. Hybrid features extracted from CTD, PseAAC, PSSM, and disorder are utilized to transform the protein sequences into feature vectors. The prediction performance of the RF method on the full features is compared with that of several state-of-the-art classifiers. The comparison results indicate that RF is an ideal choice to construct the optimal model for predicting cancerlectins. SMOTE has been demonstrated to have the effective role in the imbalanced data classification problem. To improve the prediction performance, the two-step feature selection process employed in this study can significantly remove redundant or uninformative features. Randomization test has been performed to validate the robustness of our model. Compared with some previously published methods on the same dataset using 5-fold cross-validation, the proposed method has a good capacity to identify cancerlectins. These results indicate the proposed method is a useful tool for identifying cancerlectins.

## Figures and Tables

**Table 1:** Division of the 20 natural amino acids according to different physicochemical properties.

Physicochemical properties	Group 1	Group 2	Group 3
Hydrophobicity	DEKNQR	AGHPSTY	CFILMVW
Normalized van der Waals volume	ACDGPST	EILNQV	FHKMRWY
Polarity	CFILMVWY	AGPST	DEHKNQR
Polarizability	ADGST	CEILNPQV	FHKMRWY
Charge	KR	DE	ACFGHILMNPQSTVWY
Secondary structures	AEHKLMQR	CFITVWY	DGNPS
Solvent accessibility	ACFGILVW	DEKNQR	HMPSTY

**Table 2:** Performance comparisons of different machine learning methods on the full features using 5-fold cross-validation.

Machine learning method	Sensitivity	Specificity	Accuracy	MCC
AdaBoost	0.690	0.540	0.615	0.233
Decision Table	0.681	0.540	0.611	0.223
Nearest Neighbor Analysis	0.757	0.584	0.670	0.346
Logistic Regression	0.531	0.558	0.544	0.089
Naïve Bayes	0.500	0.699	0.600	0.203
RBFNetwork	0.615	0.491	0.553	0.107
Random Forest	0.704	0.695	0.699	0.398

**Table 3:** Prediction results with and without SMOTE on the full features using 5-fold cross-validation.

Method	Sensitivity	Specificity	Accuracy	MCC
Without SMOTE	0.461	0.717	0.604	0.085
With SMOTE	0.704	0.695	0.699	0.398

**Table 4:** The prediction performance trained with the 13 optimal features and the prediction performance trained with the 13 features that are randomly selected from original features.

Method	Sensitivity	Specificity	Accuracy	MCC	AUC
Randomly selected features	0.631	0.609	0.620	0.240	0.659
Optimal features	0.779	0.717	0.748	0.497	0.787

**Table 5:** Performance comparisons with the existing methods using 5-fold cross-validation.

Method	Sensitivity	Specificity	Accuracy	MCC	Feature number
Amino Acid Composition [20]	0.680	0.642	0.658	0.32	20
Dipeptide Composition [20]	0.673	0.628	0.648	0.30	400
Split based Composition (2-part) [20]	0.663	0.642	0.651	0.31	40
Split based Composition (4-part) [20]	0.651	0.669	0.661	0.32	80
Position-Specific Scoring Matrix [20]	0.679	0.686	0.683	0.36	400
PSSM with 14 PROSITE domains [20]	0.680	0.699	0.691	0.38	414
*g*-gap dipeptides [22]	0.691	0.801	0.752	0.495	68
Our method	0.779	0.717	0.748	0.497	13

## Supplementary Materials

Supplementary 1 The final dataset that consists of 178 cancerlectins and 226 noncancerlectins.

Supplementary 2 The score for each of the features evaluated by Relief.

Supplementary 3 The detailed prediction results against different numbers of features by performing the SFS scheme.
